# Comprehensive Molecular Landscape of Cetuximab Resistance in Head and Neck Cancer Cell Lines

**DOI:** 10.3390/cells11010154

**Published:** 2022-01-04

**Authors:** Izabela N. F. Gomes, Renato J. da Silva-Oliveira, Luciane Sussuchi da Silva, Olga Martinho, Adriane F. Evangelista, André van Helvoort Lengert, Letícia Ferro Leal, Viviane Aline Oliveira Silva, Stéphanie Piancenti dos Santos, Flávia Caroline Nascimento, André Lopes Carvalho, Rui Manuel Reis

**Affiliations:** 1 Molecular Oncology Research Center, Barretos Cancer Hospital, Barretos 14784-400, Brazil; izabela.faria.tk@hotmail.com (I.N.F.G.); renatokjso@gmail.com (R.J.d.S.-O.); lsussuchi@gmail.com (L.S.d.S.); adriane.feijo@gmail.com (A.F.E.); ahlengert@gmail.com (A.v.H.L.); leticiaferro@hotmail.com (L.F.L.); vivianeaos@gmail.com (V.A.O.S.); carvalhoal@gmail.com (A.L.C.); 2 Barretos School of Medicine Dr. Paulo Prata—FACISB, Barretos 14785-002, Brazil; 3 Life and Health Sciences Research Institute (ICVS), Medical School, University of Minho, 4710-057 Braga, Portugal; d6404@med.uminho.pt (O.M.); fcncarol@hotmail.com (F.C.N.); 4 Laboratory of Molecular Diagnosis, Barretos Cancer Hospital, Barretos 14784-400, Brazil; stefipiacentis@hotmail.com; 5 3ICVS/3B’s-PT Government Associate Laboratory, 4710-057 Braga, Portugal

**Keywords:** EGFR, cetuximab, drug resistance, head and neck tumors, biomarkers, in vitro, pre-clinical

## Abstract

Cetuximab is the sole anti-EGFR monoclonal antibody that is FDA approved to treat head and neck squamous cell carcinoma (HNSCC). However, no predictive biomarkers of cetuximab response are known for HNSCC. Herein, we address the molecular mechanisms underlying cetuximab resistance in an in vitro model. We established a cetuximab resistant model (FaDu), using increased cetuximab concentrations for more than eight months. The resistance and parental cells were evaluated for cell viability and functional assays. Protein expression was analyzed by Western blot and human cell surface panel by lyoplate. The mutational profile and copy number alterations (CNA) were analyzed using whole-exome sequencing (WES) and the NanoString platform. FaDu resistant clones exhibited at least two-fold higher IC_50_ compared to the parental cell line. WES showed relevant mutations in several cancer-related genes, and the comparative mRNA expression analysis showed 36 differentially expressed genes associated with EGFR tyrosine kinase inhibitors resistance, RAS, MAPK, and mTOR signaling. Importantly, we observed that overexpression of KRAS, RhoA, and CD44 was associated with cetuximab resistance. Protein analysis revealed EGFR phosphorylation inhibition and mTOR increase in resistant cells. Moreover, the resistant cell line demonstrated an aggressive phenotype with a significant increase in adhesion, the number of colonies, and migration rates. Overall, we identified several molecular alterations in the cetuximab resistant cell line that may constitute novel biomarkers of cetuximab response such as mTOR and RhoA overexpression. These findings indicate new strategies to overcome anti-EGFR resistance in HNSCC.

## 1. Introduction

Head and neck squamous cell carcinoma (HNSCC) is the eighth most common neoplasia worldwide, accounting for 377,000 new cases and 177,000 deaths from this disease every year [1]. HNSCC can be associated with tobacco and alcohol consumption, representing 75% of all cases. However, infection with the Human Papilloma Virus (HPV) high-risk types are increasingly detected and currently considered one oncogenic driver in a subset of cases [2]. HNSCCs also harbor a high genetic heterogeneity, with mutations in tumor suppressor genes such as *TP53* and *p16INK4a* and activation of oncogenes, such as the epidermal growth factor receptor (*EGFR*) and *PIK3CA* [3,4,5].

EGFR is a transmembrane protein that belongs to the HER/ErbB family of tyrosine kinases receptors (RTK), including ErbB2/Neu/Her2, ErbB3/Her3, and ErbB4/Her4 [6]. Ligand binding triggers homo/heterodimerization with other HER phosphorylation, activating downstream signaling pathways promoting proliferation members and subsequent cell cycle differentiation, survival, angiogenesis, invasion, and metastasis in cancer [3,6]. Moreover, EGFR can translocate to the nucleus and acts as a transcriptional factor [7]. EGFR is overexpressed in 90% of all HNSCCs and correlates with poor survival and treatment outcomes [3,8].

Currently, the only anti-EGFR therapy FDA approved for HNSCC is Cetuximab [3]. Cetuximab is a chimeric monoclonal antibody that targets EGFR by competitively inhibiting their natural ligands, fostering EGFR internalization, and altering EGFR-dependent signaling [6,9]. Cetuximab demonstrated a 13% response rate in recurrent or metastatic HNSCC as monotherapy [10]. The combination of cetuximab and the first-line chemotherapy (platinum–fluorouracil) also improves response rates, progression-free survival (PFS), and overall survival (OS) (10.1 months versus 7.4 months; *p* = 0.04) in recurrent or metastatic HNSCC patients [11]. Moreover, in patients with advanced locoregionally disease, cetuximab plus radiotherapy improves OS compared with radiotherapy alone (49.0 months versus 29.3 months; *p* = 0.03) [12]. In addition, the locoregional control of HNSCC patients was 24.4 months among patients treated with cetuximab plus radiotherapy and 14.9 months among those given radiotherapy alone [12]. Despite encouraging response rates, cetuximab has shown high recurrence levels in HNSCC. Although *PIK3CA* and *RAS* mutations and *PTEN* expression were previously related as potentially biomarkers [13], the molecular mechanisms underlying cetuximab resistance remain unclear [14].

Herein, to identify new biomarkers related to the acquired cetuximab resistance in HNSCC, we developed a cetuximab-resistant in vitro model, performed an integrated molecular investigation and compared the newly acquired features with the parental cells to uncover particular genes or pathways involved in the acquired cetuximab resistance that could be related to the disease development.

## 2. Materials and Methods

### 2.1. Cell Lines and Cetuximab Resistance Model Development

FaDu cell line was obtained from American Type Culture Collection (ATCC catalog number HTB-43). The cells were maintained in Dulbecco’s modified Eagle’s medium (DMEM 1X, high glucose; Gibco, Invitrogen, Grand Island, NY, USA) supplemented with 10% Fetal Bovine Serum (FBS, Gibco, Invitrogen Grand Island, NY, USA) and 1% penicillin/streptomycin solution (P/S, Sigma-Aldrich, San Luis, MO, USA), at 37 °C and 5% CO_2_. The Department of Molecular Diagnostics, Barretos Cancer Hospital performed authentication of cells in June 2019, as previously reported [15]. Moreover, all cell lines were tested for mycoplasma through MycoAlertTm Mycoplasma Detection Kit (Lonza, Basel, Switzerland), following the manufacturer’s instructions.

The cetuximab resistance model was developed by growing the sensitive cell line FaDu in increasing (200–3200 µg/mL) sub-lethal concentrations of cetuximab (Merck, Darmstadt, Germany) in the growth medium. The starting dose was approximately the IC_50_ (inhibitory concentration, 50%) of the cell line for 72 h. The medium was then removed to allow the cells to recover for a further 72 h. This development phase was conducted for approximately eight months, and each clone was removed from the culture at a specific concentration, and then the cell viability assay was re-assessed. In the end, we obtained seven stable resistant clones. The resistant cell obtained (FaDu resistant) was cultivated in the same conditions as the parental cell line. Fadu Parental (FaDu P) was used as non-resistant control for all experiments. The stock solutions of cetuximab were prepared in phosphate-buffered saline (PBS, Sigma-Aldrich, San Luis, MO, USA) and stored at 4 °C.

### 2.2. Chromosome Preparation and G-Banding

The karyotyping analysis was performed as described previously [16]. Fadu parental and FaDu resistant were incubated with 120 ng/mL colcemid (Life Technologies–Gibco, Invitrogen Grand Island, NY, USA) for 120 min. The cells were harvested by treatment with 0.025% acutase, suspended in KCl 0.075 M solution at 37 °C temperature for 8 min, and fixed with methanol/acetic acid (3:1) five times. The G banding was obtained using 0.0125 g/mL trypsin and 4% Giemsa solution. Twenty metaphases were analyzed using the Olympus BX61, BandView 7.2.7 (Applied Spectral Imaging/Genasis), and the result was described according to the International Human Cytogenetic Nomenclature System (ISCN 2016).

### 2.3. DNA Isolation and Whole Exome Sequencing (WES) Analysis

The DNA from FaDu parental and FaDu resistant was used for WES, with the input of 1 µg on the Illumina NovaSeq™ System by a commercial company (Sophia Genetics, Switzerland). Sequence reads were aligned using human reference genome build 37 (hs37d5-decoy) applying BWA-MEM with Burrows–Wheeler Aligner version 0.7.10-789 [17]. Duplicate reads were marked with Picard-Tools 1.92 (http://broadinstitute.github.io/picard/, accessed on 19 October 2021). MuTect version 1.1.4; (http://www.broadinstitute.org/cancer/cga/mutect, accessed on 19 October 2021) and Varscan2 [18] were used to call somatic SNVs and indels parental cell line, respectively. MuTect was run using default parameters with files from COSMIC version 54 and dbSNP version 132 included as input [19,20]. We used Ensembl Variant Effect Predictor (VEP) [21] to annotate and determine tumor-specific variants’ functional effects. The results from SIFT, Polyphen-2, ClinVar were considered. It also excluded variants likely to be germline, i.e., listed in ESP6500 (http://evs.gs.washington.edu/EVS/, accessed on 19 October 2021), 1000 Genome, or ExAC [22,23]. The candidate mutations were manually curated using the Integrated Genomics Viewer (IGV) [24]. Copy number abnormalities (CNA) were identified using Nexus Copy Number version 9.0 (BioDiscovery; El Segundo, CA, USA; https://www.biodiscovery.com/products/Nexus-Copy-Number, accessed on 19 October 2021) with a default parameter for BAM ngCGH (matched) input with homozygous frequency threshold and value at 0.97 and 0.8, respectively, hemizygous loss threshold at −0.18, single copy gain at 0.18 and high copy gain at 0.6.

### 2.4. Copy Number Alterations (CNA)

CNA was analyzed using the nCounter^®^ v2 Cancer CN Assay panel (NanoString Technologies, Seattle, WA, USA). This panel counts the Copy Number Variation (CNV) of 87 involved genes commonly amplified or deleted in several cancers (www.nanostring.com/products/CNV, accessed on 19 October 2021). As a control, the genomic DNA from FaDu parental was used. First 600 ng of genomic DNA was submitted to DNA fragmentation by AluI digestion followed by a denaturation step, which yields single-stranded templates. Then, single-stranded DNA templates were submitted to hybridization with a reporter probe, which carries the color-code, and a capture probe, which allows the immobilization of the targets over the cartridge for data capture. After hybridization, samples were transferred to the nCounter^®^ Prep Station, an automated platform, to remove probes excess and probe/target complexes alignment and immobilization over the nCounter^®^ cartridge. Then, cartridges were placed in the nCounter^®^ Digital Analyzer for data capture. Raw data was captured by the nSolverAnalysis Software v4.0^®^ (NanoString Technologies) and normalized by the average of 54 probes targeting invariant regions (invariant controls). For final data estimation, CNA estimated twice the ratio of the average probe count per gene in the resistant cell line than the parental cell line as previously described [25].

### 2.5. Cell Surface Markers Screening

The cell surface markers were analyzed according to the manufacturers’ instructions. BD Lyoplate™ Human Cell Surface Marker Screening Panel was utilized (cat. 560747; BD Biosciences, Franklin Lakes, NJ, USA) containing 242 purified monoclonal antibodies and corresponding isotype controls following the manufacturer’s instructions. Briefly, 5 × 10^3^ cells were seeded in a 96-well plate in fluorescence-activated cell sorting (FACS) buffer containing 10 mg/mL DNAse. Primary antibody incubation was carried out in 27 mL volume for 30 min on ice, followed by two washes in FACS buffer washes. Next, cells were incubated with biotinylated secondary antibodies (goat anti-mouse 1:200, goat anti-mouse 1:200). The cells were fixed in 1000 µL of 4% paraformaldehyde in 1× PBS and washed in FACS buffer washes. FACS analysis gating allowed the elimination of debris. The analysis was performed using the BD ACCURI™C6 flow cytometer using at least 10,000 events per well.

### 2.6. Western Blot, Human RTK, Subcellular Fraction Separation, and Cytokines Arrays

To assess the effect of cetuximab resistance in the intracellular signaling pathways and RTKs, the cells were cultured in DMEM 10% FBS in T25 culture flasks, which grew to 85% confluence and then serum starved for 2 h. The cells were washed and scraped in cold PBS and lysed in a buffer containing 50 mM Tris (pH 7.6–8), 150 mM NaCl, 5 mM EDTA, 1 mM Na3VO4, 10 mM NaF, 10 mM sodium pyrophosphate, and 1% NP-40 and protease cocktail inhibitors. Western blot analysis was performed using a standard 10% sodium dodecyl sulfate-polyacrylamide gel electrophoresis, loading 20 μg of protein per lane. All the antibodies were used as recommended by the manufacturer. All the antibodies were used as recommended by the manufacturer (Supplementary Table S3). We used the Cell Fractionation Kit (cell signaling; #9038) for subcellular fraction separation according to the manufacturer’s instructions. Concerning the RTK phosphorylation assessment, a proteome human RTK Phosphorylation Antibody Array (ab193662; Abcam, Cambridge, UK) and Human XL Cytokine Array Kit (ARY022; R&D systems, Minneapolis, MM, USA) was used according to the manufacturer’s instructions. A total of 500 μg of fresh protein lysates was briefly incubated overnight at 4 °C with nitrocellulose membranes dotted with duplicated spots for 71 anti-RTK, 102 anti-Cytokines, and control antibodies. Bound phospho-RTKs and cytokines were incubated with a pan anti-phosphotyrosine-HRP antibody for 2 h at room temperature. Blot detection was performed by chemiluminescence (ECL Western Blotting Detection Reagents, RPN2109; GE Healthcare, Piscataway, NJ, USA) in ImageQuant LAS 4000 mini (GE Healthcare, Chicago, IL, USA). The bands were quantified by densitometry using the Image J software (1.49v). For subcellular fraction assay first, the total protein was normalized by the loading control (β-actin) or by loading control for each cellular compartment (lamin B1 for nuclear and FADD for cytoplasm). Additionally, relative ratios (phospho/total) were determined.

### 2.7. mRNA NanoStringTM Analysis

Gene expression analysis on FaDu parental and FaDu resistant was performed using the NanoString nCounter PanCancer Pathways panel (730 gene transcripts) according to the manufacturers’ instruction (NanoString Technologies, Seattle, WA, USA). This panel assesses thirteen canonical pathways (Notch, Wnt, Hedgehog, Chromatin modification, Transcriptional Regulation, DNA Damage Control, TGF-beta, MAPK, STAT, PI3K, RAS, Cell Cycle and Apoptosis). Briefly, 100 ng aliquots of RNA were hybridized with probe pools, hybridization buffer, and codeset reagents in a total volume of 30 μL and incubated at 65 °C for 20 h. After codeset hybridization overnight, the samples were washed and immobilized to a cartridge using the Nanostring nCounter Prep Station (NanoString Technologies, Seattle, WA, USA) for 4 h. Finally, the cartridges containing immobilized and aligned reporter complexes were scanned in the nCounter Digital Analyzer (NanoString Technologies), and image data were subsequently generated using the high-resolution setting. Quality control assessment of raw NanoString gene expression counts was performed with nSolver Analysis Software version 4.0 and the default settings (NanoString Technologies, Seattle, WA, USA). Quantile normalization and differential expression were performed within the NanoStringNorm package (v1.2.1.1) [26] in the R statistical environment (v3.6.3). The normalized log2 mRNA expression values were used for subsequent data analysis. Genes with fold change (FC) ≥ ±2 and *p* < 0.01 were considered significant. Heatmap with hierarchical clustering of differentially expressed genes was built in the ComplexHeatmap package (v2.0.0) [27].

### 2.8. Expression Microarray

The expression profile of the FaDu cells was analyzed by microarray slides Gene Expression Microarray, 4 × 44 K (Agilent Technologies, Santa Clara, CA, USA) in dual-color methodology (Cy3 and Cy5), following the manufacturer’s recommendations. Total RNA (500 ng) with the addition of control RNA (spike-in) were carried out to reverse transcription with the aid of the enzyme MMLV-RT (Maloney Murine Leukemia Virus—Reverse Transcriptase). Subsequently, nucleotides marked with the fluorochromes cyanine 5 (Cy5) were added to the T7 polymerase enzyme to the parental and resistant cells cDNA. The respective cDNAs were purified with commercial columns and then hybridized on the slides for 17 h at a constant temperature of 65 °C in a hybridization oven (Agilent Technologies). Finally, according to the manufacturer’s recommendations, the hybridized slides were washed in Wash Buffer 1 and Wash Buffer 2. The slides were scanned at 550 nm (green spectrum—-Cy3) and 640 nm (red spectrum-Cy5) on the Agilent Scanner Surescan (Agilent Technologies). Data extraction and quality control were performed using the Feature Extraction software, version 12 (Agilent Technologies). Oligonucleotides were identified by the customized protocol GE2_107_Sep09. After quantification, the raw data means (gMeanSignal) were selected to subtract the background means (gBGMeanSignal) used for future analyses. The expression data were analyzed in an R environment, version 4. The limma package was used to remove positive and negative controls, values that overlapped the background, and data conversion on a logarithmic scale for the inclusion of data, identification of flags, and generation of expression matrices. Then, the data were normalized by the quantile methodology, and the moderated t test was applied, followed by false-discovery rate (FDR) correction. The category that presented at least three genes and a classification *p* ≤ 0.05 was considered significant after Benjamini–Hochberg correction.

### 2.9. Sanger Sequencing

The analysis of ROS1 mutation (c.6341A>G) was performed by PCR followed by direct Sanger sequencing, as described previously [28]. Briefly, using a specific pair of primers (forward 5′-3′: AGCATTACTCTGTGTCCCGT; reverse 5′-3′: AGGGATCTGGCAGCTAGAAA), the target region was amplified by PCR using a Veriti PCR thermal cycler (Applied Biosystems, Foster City, CA). We used an initial denaturation at 95 °C for 1 min followed by 35 cycles of 95 °C denaturation for 30 s; specific annealing temperature was for 30 s and the 72 °C elongation phase for 30 s, followed by a 72 °C final elongation for 2 min. Amplification of PCR products was confirmed by gel electrophoresis. Sequencing PCR was performed using the BigDye Terminator v3.1 Cycle Sequencing Kit (Applied Biosystems, Waltham, MA, USA) and a 3500 xL Genetic Analyzer (Applied Biosystems, Waltham, MA, USA).

### 2.10. Cell Viability Assay

The cell viability was performed by MTS (Promega, Madison, WI, USA) as previously described [15]. To determine the IC_50_ values, cells were seeded into 96-well plates at a density of 5 × 103 cells per well and allowed to adhere overnight in DMEM 10% FBS. Subsequently, the cells were treated with increasing concentration of cetuximab (0, 20, 30, 50, 100, 200 e 250 µg/mL) diluted in DMEM-0.5% FBS for 72 h. The results were expressed as mean viable cells relative to PBS alone (considered 100% viability) ± SD. The IC_50_ concentration was calculated by nonlinear regression analysis using GraphPad Prism software version 7. The assays were performed in triplicate at least three times.

### 2.11. Morphological Data

The images of the cell lines’ morphological changes after treatment with cetuximab were obtained in Olympus XT01 (Olympus, Shinjuku, Tokyo, Japan) microscope at 100× magnification.

### 2.12. Wound Healing Migration Assay

The cells were seeded in six-well plates and cultured in DMEM-10%FBS to at least 95% of confluence. Monolayer cells were washed with PBS and scraped with a plastic 100 μL pipette tip. The “wounded” areas were photographed by phase-contrast microscopy after 0, 24, 48, and 72 h. The following formula calculated the relative migration distance: percentage of wound closure (%) = 100(A − B)/A, where A is the width of cell wounds before incubation, and B is the width of cell wounds after incubation [29].

### 2.13. Colony Formation Assay Anchorage-Dependent

FaDu parental and FaDu resistant cells were seeded at a density of 5 × 10^3^/well in six-well plates and maintained in a culture medium for 8–10 days at 37 °C. Finally, colonies were stained with 0.125% crystal violet solution. Colonies larger than 50 cells were photographed under the light microscope Eclipse 2200 (Nikon, Minato, Tokyo, Japan), and the number of colonies was analyzed by the open-source software CFU (Plos One—http://opencfu.sourceforge.net/, accessed on 20 October 2021) [30]. The results represent the mean of at least three independent experiments.

### 2.14. Adhesion Assay

Adhesion of FaDu parental and FaDu resistant cell lines was evaluated as described previously [31]. Briefly, a 96-well plate was coated for 24 h with PBS, BSA (bovine serum albumin, 10 μg/mL, Sigma-Aldrich), Matrigel^®^. Matrigel^®^ was diluted at 1:10 in PBS1X. The next day the excess liquids were removed, and the plates were incubated with 100 μL/well of 0.1% BSA for two h and washed with PBS. Then, 6 × 10^3^ cells of FaDu parental and FaDu resistant cells were added to each well in a serum-free medium and incubated at 37 °C in a 5% CO_2_ humidified atmosphere for 2 h. The non-adherent cells were rinsed off, and the remaining cells were fixed with 10% trichloroacetic acid (TCA), stained with crystal violet and quantified using an ELISA reader at 540 nm.

### 2.15. Immunofluorescence Analysis

Epidermal growth factor receptor (EGFR) was stained as described [32], 2.5 × 10^4^ cells were seeded in 12-well plate in DMEM 10%. The next day, cells were washed in PBS and permeabilized with 0.3% Triton X-100 for 10 min. Cells were incubated with primary antibodies, Goat anti-EGFR (1:1000, Dako, Santa Clara, CA, USA), and Rabbit anti-phospho-mTOR (1:1000, cell signaling) for 24 h at 4 °C. Cetuximab (CTX) was detected using anti-human IgG Fc antibody (1:1000; R&D systems) for 24 h at 4 °C. After the wash step with PBS1X, cells were incubated for 1 h at room temperature with Alexa 560 labeled anti-goat secondary antibodies (1:400, Invitrogen). Sections were then stained with Hoechst (Thermo Scientific, Waltham, MA, USA) and washed with PBS three times. The cells were analyzed using fluorescence microscopy (Olympus, Fluoview FV10, Shinjuku, Tokyo, Japan) at 600× magnification.

### 2.16. Statistical Analysis

Single comparisons between the conditions studied were made using Student’s t test, and the differences between the groups were tested using variance analysis. The statistical analysis was performed using GraphPad Prism version 8.4.3. The level of significance in all statistical analyses was set as *p* < 0.05.

## 3. Results

### 3.1. Cetuximab-Resistance Model Establishment and Characterization

**Figure 1:**
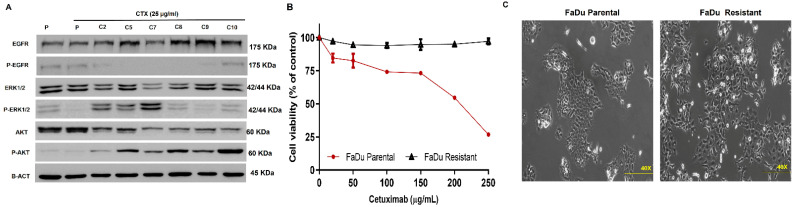
Cetuximab resistant model establishment and characterization. (**A**) EGFR signaling of parental and cetuximab-resistant clones after cetuximab resistant model establishment. (**B**) Cell viability assay of parental and resistant clone upon cetuximab exposition in 72 h. (**C**) Cell morphology of parental and resistant cells P: FaDu parental; R: FaDu resistant; C: Clone. The images were acquired in 10× magnification.

To establish an HNSCC cetuximab-resistant in vitro model, FaDu cells were treated with increasing cetuximab doses for eight months, leading to the establishment of seven resistant clones. All resistant clones decreased EGFR phosphorylation levels upon cetuximab treatment and demonstrated at least a two-fold higher IC_50_ ranging from 400 µg/mL to 5320 µg/mL compared with parental cells (Figure 1A and Supplementary Table S1). Interestingly, clone C5 exhibits a profile of resistance to ERK and AKT inhibition despite demonstrating complete EGFR inhibition (Figure 1A). For this response profile, we chose this clone for further analysis. C5 clone demonstrated higher IC50 fold-change (17-fold, Supplementary Table S1) compared to FaDu parental, as we can see in the cell viability profile (Figure 1B). No other significant changes in others tyrosine kinases receptors were observed in the protein array (Supplementary Figure S1A). Besides, the C5 clone depicted a marked alteration of cellular morphology, exhibiting a spindle shape and scattering profile compared to the epithelial morphology of parental cells, suggesting loose cell–cell interaction (Figure 1C).

### 3.2. Cetuximab Resistance Is Associated with Chromosomal Abnormalities

**Figure 2:**
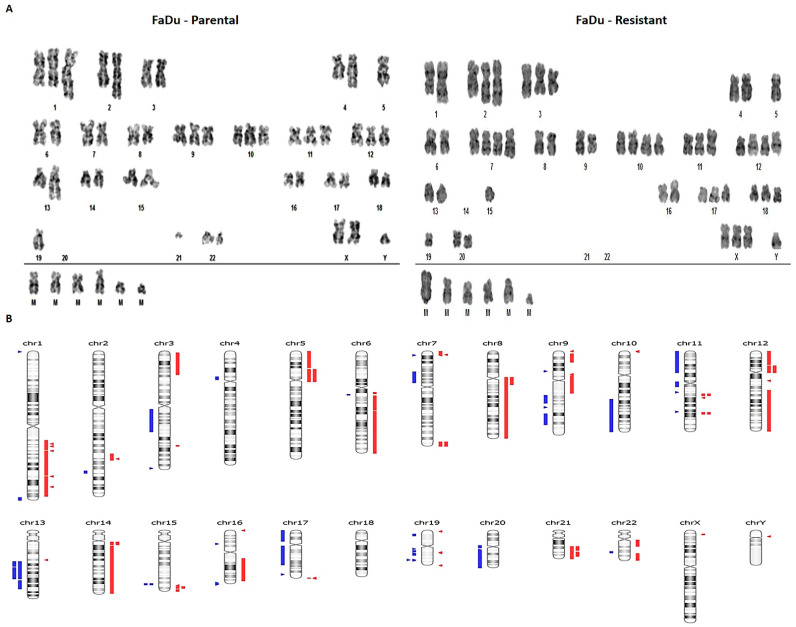
Karyotypes and Copy number alterations (CNA) overview of FaDu parental and FaDu resistant cells. (**A**) Representative metaphase of FaDu parental and FaDu resistant cells. (**B**) Overview of the CNAs found in FaDu resistant cells in comparison with FaDu parental cells. In red gain and in blue deletions. M: marker chromosome.

To evaluate cetuximab resistance’s molecular impact, we further performed a detailed genomic and transcriptomic profile comparison between parental and FaDu C5 resistant cells. FaDu parental and resistant cell lines’ karyotyping analysis revealed severe aneuploidy, with 51–57 chromosomes, with 11 numerical alterations and several chromosomal structural changes (Figure 2A). The parental cells presented hyperdiploid and composite karyotype with 51 to 53 chromosome figures, with several aberrations such as trisomy of chromosomes 1, 2, 3, 6, 7, 8, 9, 10,11, 12, 16, and 17, monosomy of chromosomes 5, 13, 19, 20, 21 and 22, and tetrasomy of chromosome 18. The resistant cell line had a hyperdiploid and composite karyotype with many chromosomes, ranging from 52 to 56, showing several alterations such as trisomy of X, 1, 2, 3, 6, 8, 11, 16, 17, and 18 chromosomes, monosomy of chromosomes 4, 5, 13, 14, 19, 20, 21 and 22, and tetrasomy of chromosomes 7, 9, 10 and 12 (Supplementary Table S2).

The chromosomal or copy number aberrations (CNA) were also evaluated by whole-exome sequencing (WES) data and CNA Nanostring panel of 87 genes. The WES showed 31 chromosomal regions with CNA associated with cancer by Cancer Genome Interpreter (CGI) and Nexus (Figure 2B). There were 15 amplified regions, with one presenting high amplification, harboring genes such as RHOA, KRAS, MYB, MAP3K5, BCL2L2, and YAP1 (Table 1). Deletions were found in 15 regions, harboring genes such as TP53, PTEN, WT1, BRCA1, MAP2K4, and NF1. These data are in accordance with karyotyping analysis, where we also found amplification of chromosomes 7 and 12, containing the same amplified genes found in CNA analysis such as KRAS, MDM2, HMGA2, SHH, CCDND2, and FRS2. Many of the altered loci identified are related to MTOR-PI3K-AKT and MAPK proliferation signaling pathways. Other altered regions harbored genes mainly related to the DNA repair process, apoptosis, and transcriptional factors. All genes reported as tumor drivers by Cancer Genome Interpreter (CGI) [33] are shown in Table 1.

### 3.3. Differential Gene Expression and Mutation Profile

**Figure 3:**
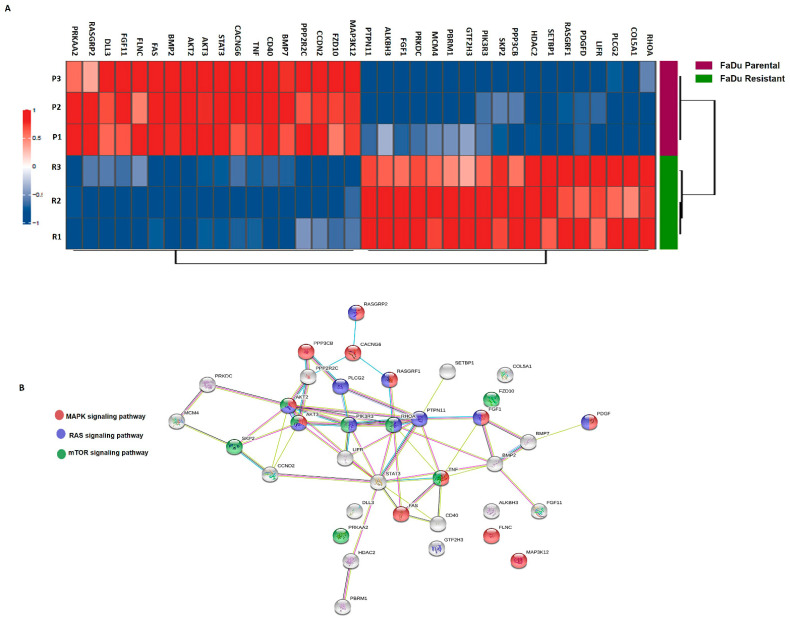
Molecular characterization after cetuximab-acquired resistance. (**A**) Heatmap of genes altered in FaDu parental and FaDu resistant cells. In red are represented the overexpressed genes, and in blue the downregulated genes. FaDu parental is shown in purple and FaDu resistant in green. (**B**) Genetic interaction network associated with cetuximab resistance on the STRING database. In this figure, each circle represents a protein (node), and each connection represents a direct or indirect connection (edge). Line color indicates the type of interaction evidence: purple—experimental evidence, light blue—curate database, black—co-expression, pink—experimentally determined, yellow—text mining, dark blue—gene co-occurrence (MAPK associated genes are shown in red, RAS associated genes are shown in blue, and mTOR signaling-related genes are shown in yellow. *p*. adjusted <0.01; FC ≥ 2.

The differential transcriptomic profile of FaDu parental and resistant cells was evaluated using the NanoString PanCancer Pathways Panel. Of the 730 genes evaluated, 36 were differentially expressed, with 18 genes upregulated (RHOA, COL5A1, PLCG2, LIFR, PDGFD, RASGRF1, SETBP1, HDAC2, PPP3CB, SKP2, PIK3R3, GTF2H3, PBRM1, MCM4, PRKDC, FGF1, ALKBH3, PTPN11) and 18 downregulated (MAP3K12, FZD10, CCND2, PPP2R2C, BMP7, CD40, TNF, CACNG6, STAT3, AKT3, AKT2, BMP2, FAZ, FLNC, FGF11, DLL3, RASGRP2, PRKAA2) (Figure 3A). Next, we performed an analysis of the functional connections among the proteins encoded by the 36 genes differentially expressed by the STRING database (v11.0) [34]. In particular, FaDu resistant cells, 28 of 36 genes, had at least two connections (Figure 3B). Notably, 9 out of 28 genes demonstrated stronger evidence of connection types centered around them. (PTPN11, RHOA, PLCG2, PPP2R2C, PP3CB, AKT2, AKT3, PIK3R3, and STAT3). These findings suggest the involvement of MAPK signaling, Ras signaling, mTOR-PI3K-AKT signaling in cetuximab resistance, following our previous CNA results.

Furthermore, both parental and resistant FaDu cells’ whole-exome sequencing unveiled 394 mutations present only in the resistant cells. Mutations with important biological significance are summarized in Table 2, including driver genes such as NOTCH1, EPHA2, TSC1, ALK, and ROS1 (Supplementary Figure S1B,C). ROS1 c.6341A>G demonstrated a variant allele frequency (VAF) of 24% in resistant cells when compared with parental cells (Supplementary Figure S1B). Due to the potential therapeutic impact of ROS1 alterations, this mutation was further validated by Sanger sequencing (Supplementary Figure S1C).

### 3.4. Differential Protein Profile

**Figure 4:**
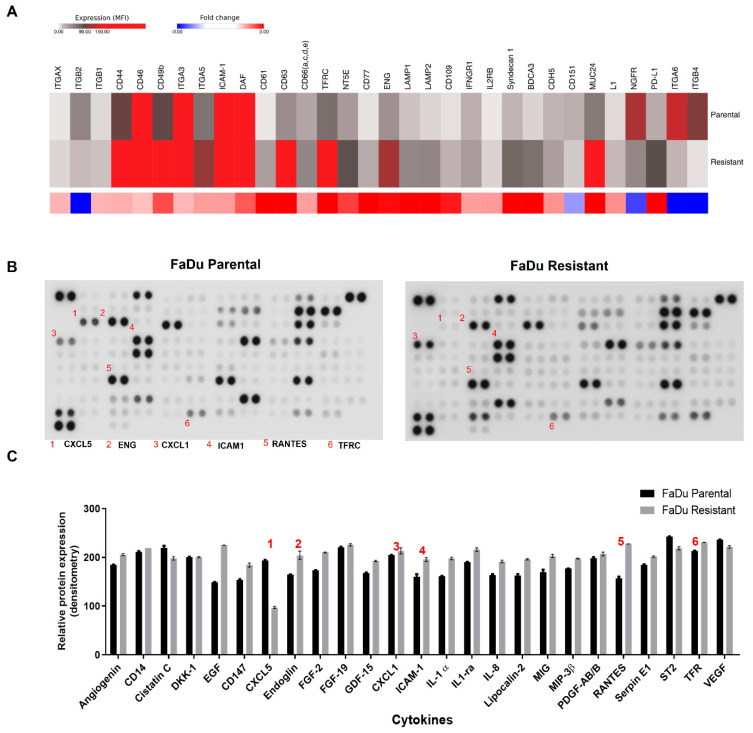
Cell surface markers and cytokines profile expression in FaDu parental and FaDu resistant cells. (**A**) Representation of cell-surface markers expression in FaDu parental and FaDu resistant cells. *p*. adjusted < 0.01. (**B**) Representative images of Cytokines protein array in FaDu parental and FaDu resistant cells. (**C**) Bars demonstrated the cytokines differential expression in FaDu cells.

Parental and resistant cells were analyzed by flow cytometry using BD lyoplate™, an extensive screening panel of 242 human cell surface proteins. We found 131 cell surface markers differentially expressed between parental and resistant cells. Among them, we found the overexpression of some mesenchymal stem cells markers (MSCs) in resistant cells, such as CD44, intercellular cell adhesion molecule 1 (ICAM-1), endoglin (ENG), programmed death-ligand 1 (PD-L1), lysosomal-associated membrane protein 1 (LAMP1) and lysosomal-associated membrane protein 2 (LAMP2) (Figure 4A). To validate the BD lyoplate™ results, some proteins were assessed by the human cytokines array protein and corroborated the overexpression of several markers such as ICAM1, TFRC, and ENG (Figure 4B,C).

### 3.5. EGFR Nuclear Translocation and mTOR Overexpression Are Present in Cetuximab-Resistant Cells

**Figure 5:**
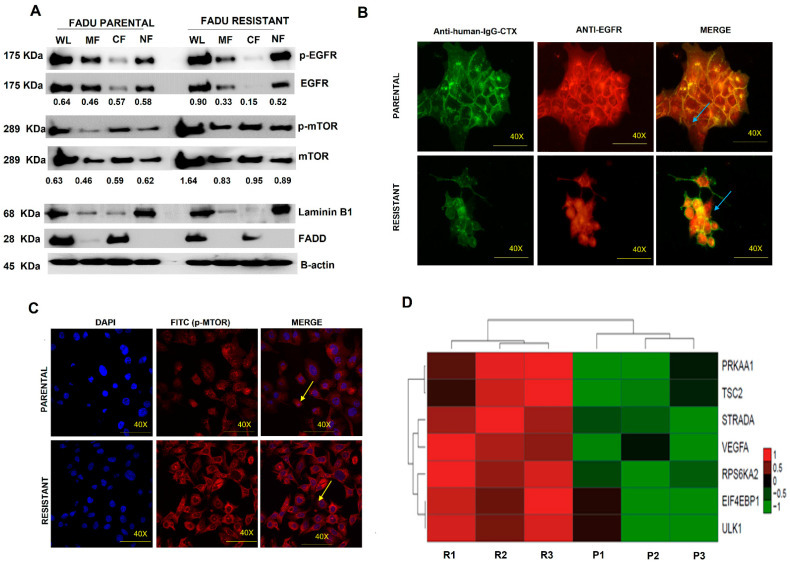
EGFR-FITC+ internalization and mTOR-FITC+ expression in FaDu parental and FaDu resistant cells. (**A**) Subcellular protein fractionation assay for EGFR detection in parental and resistant cells. (**B**) p-EGFR nuclear translocation in resistant cells by Immunofluorescence assay. (**C**) p-mTOR immunofluorescence assay in parental and resistant cells. (**D**) mTOR-related genes differentially expressed in FaDu parental and FaDu resistant by microarray of expression. DAPI (Hoescht) staining in blue. p-EGFR-FITC+ p-mTOR-FITC+. Arrows indicate EGFR-FITC+ and p-mTOR-FITC+ localization. The images were acquired in 40× magnification. The number under the bands represented relative ratios (phospho/total).

We further evaluated whether cetuximab resistance is associated with EGFR nuclear translocation. The resistant cells showed loss of EGFR in the plasma membrane (membrane fraction—MF; *p* < 0.01) and cytoplasm (cytoplasm fraction—CF; *p* < 0.01) compared with the parental cell line (Figure 5A). The EGFR nuclear translocation in resistant cells was observed by Western blot (Figure 5A) and confirmed by immunofluorescence, where we observed the presence of EGFR in the perinuclear region in resistant cells instead of the plasma membrane as found in parental cells (Figure 5B).

Since mTOR expression has been associated with cetuximab response and EGFR nuclear translocation [35], we next evaluated the mTOR expression in our resistant model. We observed an increase in mTOR protein levels in FaDu resistant cells compared to parental cells (Figure 5A; *p* < 0.01), which were also confirmed by immunofluorescence (Figure 5C). Moreover, these protein results were corroborated by our gene expression profile analysis, which showed overexpression of several genes of the mTOR pathway (Figure 5D).

### 3.6. Increased Aggressiveness Phenotype and Differential Expression of Epithelial–Mesenchymal Transition Markers (EMT) in Cetuximab Resistant Cells

**Figure 6:**
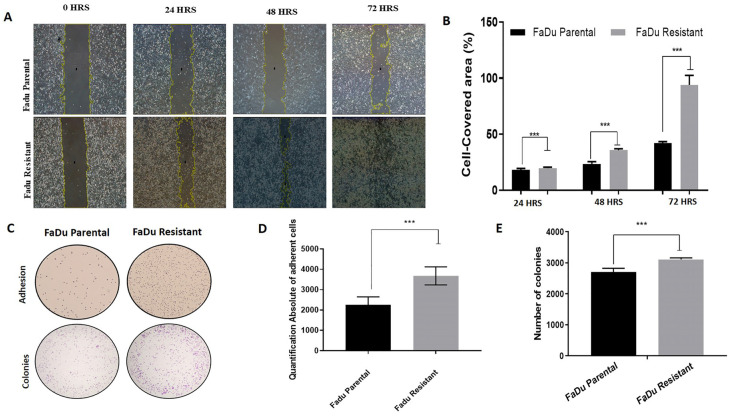
Malignant phenotype acquired after cetuximab resistance establishment. (**A**) Representative images of wound healing assay of FaDu parental and FaDu resistant cell lines in 24, 48, and 72 h. The yellow lines represent the distance between both edges of the wound; Scale bars, 200 µm; (**B**) Migration rates of FaDu parental and FaDu resistant cells in a wound-healing assay; (**C**) Representative images of adhesion and clonogenic assay for parental and resistant cells; (**D**) The absolute number of adherent cells; (**E**) The absolute number of colonies in clonogenic cell assay for anchorage-dependent in parental and resistant cells. (*** *p* < 0.0001). The images were acquired in 10× magnification.

We also performed functional assays to evaluate phenotype changes caused by cetuximab resistance. First, we evaluated the migration capability, which showed that resistant cells migrate at a higher rate than the parental cells, independent of time, assessed by wound-healing assay (Figure 6A,B). Moreover, resistant cells showed higher adhesion by protein-based assay than parental cells (Figure 6C,D). The proliferation capacity of parental and resistant cells was assessed using the clonogenic assay. There was a significantly higher fraction of surviving colonies of resistant cells relative to parental cells (Figure 6C,E). These results suggest the higher aggressive phenotype of cetuximab-resistant compared with parental cells.

**Figure 7:**
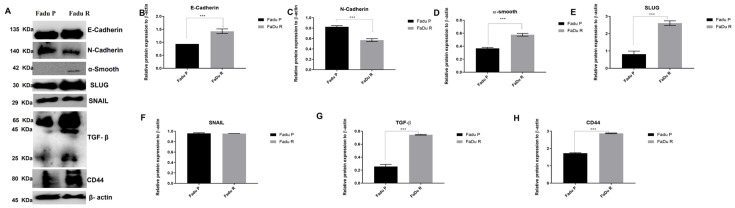
Epithelial–mesenchymal transition (EMT) markers expression in FaDu parental and FaDu resistant cells. (**A**) Representative images of EMT proteins detected in Western blot assay in parental and resistant cells. (**B**) E-cadherin densitometry. (**C**) N-cadherin densitometry. (**D**) α-smooth densitometry. (**E**) Slug densitometry. (**F**) Snail densitometry (**G**) TGF-β densitometry (**H**) CD44 densitometry Data are presented in fold-change in comparison with FaDu parental. Fadu p: FaDu parental; FaDu R: FaDu resistant. (*** *p* < 0.001).

Epithelial–mesenchymal transition (EMT) is a highly conserved cellular process that involves mesenchymal and stem cell signatures, usually presented in tumor progression, including metastasis, therapy resistance, and disease recurrence [36]. Previous studies have shown that the expression of the stemness marker CD44 is increased during EMT, and increased levels of mTOR could modulate the EMT by TGF-β [37,38]. Thus, we further evaluated EMT markers and showed decreased N-cadherin expression and increased expression of Slug, TGF-β, and CD44 in the FaDu resistant cells (Figure 7). Our previous findings of a fibroblast-shaped cell (Figure 1C), as well as overexpression of CD44, mTOR protein, and mTOR-related genes (Figure 4A and Figure 5A), corroborate with these findings.

## 4. Discussion

Targeted therapies are the key to the personalized treatment of cancer patients [39]. Herein, using an in vitro model of HNSCC, we performed a comprehensive evaluation of the molecular profile and biological mechanisms of cetuximab resistance using a long-term cetuximab exposure model. We showed that a cetuximab acquired-resistance model was successfully established and demonstrated that the overexpression of mTOR-PI3K-AKT related genes and the acquired mesenchymal and stem cell signatures might be potentially novel cetuximab-resistance (acquired) biomarkers.

Using an in vitro model to create a cetuximab resistant cell line by incremental dose exposition, we found 17-fold-higher IC_50_ for the resistant cell line, similar to the previous study conducted in HNSCC cell lines that showed a 10-fold IC_50_ for cetuximab [40]. We exposed the FaDu cell line for eight months in incremental doses to obtain cetuximab-acquired-resistant cells. Some studies have reported a resistant phenotype in 6 or 7 months at 100 and 5µg/mL of cetuximab or even in periods of shorter than 15 days [41,42,43]. We believe that resistance models based on long exposure times generate a persistent resistance to cetuximab.

We observed several chromosomal numerical aberrations with a gain of chromosomes 3, 7, and 12. Some of these alterations are known to be frequent in HNSCC, such as the gain of chromosome 7, as well as its tetrasomy [16]. Moreover, we observed specific CNA in several gene loci associated with gene overexpression in the cetuximab resistant cells, including *KRAS*, *RHOA*, *PTPN11*, *GTF2H3*, *IKZF1*, *PIK3R3*, *PLCG2*, *PDGFD*, and *RASGRF1*. In addition, we found a loss in *PTEN*. KRAS activating mutations and loss of PTEN protein expression was previously reported as a predicted biomarker of cetuximab resistance that lead to mTOR/PI3K/AKT pathway activation [13,44]. Taken together, these results suggest that where KRAS mutation is a biomarker of cetuximab response in colorectal cancer [45], in HNSCC, *KRAS* amplification may be a predictive marker of cetuximab resistance.

In our study, RHOA was the most overexpressed gene in resistant cells. We highlighted that RhoA has not been described for HNSCC as cetuximab predictive factor previously. RHOA belongs to the Rho GTPases family, which is also involved in the RAS and mTOR-PIK3-AKT signaling and controls all aspects of cellular motility and invasion, including cellular polarity, cytoskeletal organization, and transduction of signals [46,47]. Recently, Pan and coworkers reported that dysregulation of Rho GTPases, particularly Rho-A, Rho-C, and Rac2, resulted in an aggressive HNSCC phenotype [47]. Rho-A expression may indicate a poor prognosis due to the high probability to irinotecan and doxorubicin resistance in colorectal cancer [48,49]. Furthermore, Rho-A has been described to modulate the EMT by TGF-β and mTOR [50,51]. EMT is a cellular transformation process in which epithelial cells lose epithelial polarity and intercellular adhesiveness, gaining migratory potential and acquire mesenchymally and stem cell signatures [52]. In the current study, cetuximab-resistant cells showed a morphological change and increased levels of TGF-β, SLUG, and CD44, similar to EMT cell activation. We also observed an increase in adhesion rates in FaDu resistant and increased levels of mTOR, and CD44, essential regulators of EMT and metastasis [38,50,53]. Overall, this dataset reinforces that the EMT transition phenotype and overexpression of Rho-A are involved in HNSCC cetuximab resistance.

Another important therapy resistance mechanism is associated with cancer stem cells (CSCs) [54]. Our study observed a significant difference in mesenchymal stem cell markers and cell surface markers in FaDu resistant cells compared to parental cells such as CD44, ICAM-1, ENG, PD-L1 LAMP1, and LAMP2. CD44 is an important marker of CSC’s in carcinomas, and it plays a role in the mediation of resistance to drug therapy, including anti-EGFR inhibitors [55,56]. In the HNSCC context, cancer stem cells are responsible for treatment failure in which CD44 has been reported to represent a candidate of resistance marker [57]. Moreover, it has been demonstrated that CD44 regulates EGFR, activating the mTOR-PI3K/Akt signaling pathway [58].

Additionally, in our resistance model, PD-L1 (CD274) amplification/overexpression may involve the adaptive immune resistance to cetuximab. PD-L1 upregulation was associated with a higher risk for nodal metastasis at diagnosis, overall tumor-related death, and recurrence in HNSCC and associated with TKI’s resistance in lung cancer [59,60]. Importantly, it can be suggested that immune checkpoint blockers, such as pembrolizumab, which targets PD-L1, could activate an immune response and overcome the cetuximab response. Alterations of the EGFR signaling cascade are known factors influencing cetuximab response [61]. We observed a decreased EGFR phosphorylation in the resistant cells, despite the absence of variation of total EGFR and the upregulation of genes involved in the mTOR/PI3K/AKT pathway. In concordance with our results, Lida et al. showed constitutive activation of the mTOR/PI3K/AKT signaling axis in cetuximab resistant lung cell lines [62]. The upregulation of the mTOR/PI3K/AKT pathway was also reported using UM-SCC-6R cetuximab-resistant cells that demonstrated EGF-independent signaling [43]. Conversely, it was previously reported that the mTOR/PI3K/AKT pathway’s constitutive activation leads to EGFR trafficking [63]. Nuclear EGFR translocation is reported to be related to anti-EGFR therapy resistance [64]; thus, our results corroborate this hypothesis.

As mentioned, the mTOR pathway seems to play a significant role in cetuximab resistance in HNSCC. A recent study associated MTOR overexpression in the TKI inhibitors resistance process, demonstrating that stress-induced mutagenesis contributes to adaptive evolution in cancer for drug resistance [65]. Our study found an increase in mTOR levels in whole cellular compartments, including in the membrane, cytoplasm, and mainly in the nucleus of cetuximab-resistant cells. Besides, we also identified an upregulation of genes involved in mTOR signaling, namely RHOA, PIK3R3, SKP2, and a frameshift mutation in IRS2 that was not present in the parental cell line. IRS2 acts as a protein scaffold that activates the phosphoinositide 3-kinase (PI3K)/AKT/mTOR pathway, leading to proliferation and migration [66]. Thus, the inability of IRS2 to respond to negative feedback signals could contribute to higher PI3K/mTOR activity associated with cetuximab resistance in HNSCC. Of note, IRS2 mutations were recently associated with invasion in pleomorphic invasive lobular carcinoma [67]. Overall, mTOR-PIK3-AKT family members’ overexpression could be involved with cetuximab resistance, and combination regimens with drugs that block mTOR may overcome cetuximab resistance in HNSCC.

We also found a ROS1 variant (c.6341A>G) associated with resistant cells. ROS1 was previously described as regulated and associated with metastasis to lung and lymph nodes in oral squamous cell carcinoma [68]. Of note, ROS1 mutations were associated with response to anti-ROS1 inhibitors, such as Lorlatinib in pancreatic cancer [69]. Recently, Davies and coworkers demonstrated a compensatory mechanism of growth control involving ROS and EGFR, where cells resistant to ROS1 inhibition have been resensitized by inhibiting EGFR by a switch between these pathways [70]. Thus, co-targeting of ROS1 and EGFR could potentially offer an effective option to overcome cetuximab resistance. Further validation in other cell line models and in head and neck patients submitted to cetuximab are warranted to consolidate these findings.

## 5. Conclusions

In summary, this is the first report to describe the molecular basis of cetuximab resistance in HNSCC. Our study suggests that the development of cetuximab resistance in HNSCC is a complex mechanism that involves an increased number of genetic aberrations and protein dysregulation. The cetuximab acquired–resistant model was successfully established and demonstrated that the overexpression of the RhoA-mTOR-PIK3-AKT pathway and stem/mesenchymal phenotype are potentially novel cetuximab-resistance alterations. Combining regimens between cetuximab and RhoA-mTOR-PIK3-AKT inhibitors appear to be an excellent strategy to overcome cetuximab resistance. The clinical benefit from these molecules’ inhibitors should be evaluated in clinical trials, especially in a salvage setting with patients who have acquired resistance to cetuximab.

## Figures and Tables

**Table 1:** Copy number alterations differentially found in FaDu resistant compared with FaDu parental cell line by whole exome sequencing and Nanostring platform.

Chromosome	Event	Cytoband	Cancer Gene	Driver Statement by CGI
chr 17	Deletion	p13.3–p11.2	MAP2K4	known in: PA; BRCA; COREAD
chr 13	Deletion	q31.1–q32.2	GPC5	predicted passenger
chr 20	Deletion	q11.22–q13.33	EEF1A2	predicted passenger
chr 17	Deletion	p13.3–p11.2	MAPK7	predicted passenger
chr 17	Deletion	p13.3–p11.2	TP53	known in: BCL; THYM
chr 11	Deletion	p15.5–p12	WT1	known in: WT; DSRCT
chr 10	Deletion	q22.3–q26.3	PTEN	known in: G; PRAD; ED; CM; TH; BRCA; L; OV; PA
chr 7	Deletion	p14.3–p12.1	IKZF1	known in: ALL; DLBCL
chr 10	Deletion	q22.3–q26.3	SUFU	known in: MB
chr 16	Deletion	q24.3	FANCA	known in: AML; LK; PRAD
chr 17	Deletion	p13.3–p11.2	FLCN	known in: TH
chr 17	Deletion	q11.1–q23.1	NF1	known in: NF; G; MPN; CM; PLEN; HNC; SG; LK
chr 17	Deletion	q11.1–q23.1	SUZ12	known in: CANCER
chr 17	Deletion	q11.1–q23.1	BRCA1	known in: OV; BRCA
chr 12	High Amplification	p12.1–q11	KRAS	predicted driver
chr 14	Amplification	q12–q32.33	NKX2-1	known in: NSCLC
chr 14	High Amplification	q11.2–q12	BCL2L2	predicted passenger
chr 5	High Amplification	p13.3–q11.2	SKP2	predicted passenger
chr 22	Amplification	q11.1–q12.1	CRKL	predicted passenger
chr 11	High Amplification	q22.1–q22.3	YAP1, BIRC2	predicted passenger
chr 7	High Amplification	q36.1–q36.3	SHH	predicted passenger
chr 6	Amplification	q16.2–q27	MYB	predicted driver
chr 6	Amplification	q16.2–q27	MAP3K5	predicted passenger
chr 1	Amplification	q32.1–q32.2	MDM4	known in: GBM; BLCA; RB; S
chr 12	Amplification	q14.3–q24.33	MDM2, HMGA2	known in: S; G; COREAD; LIP
chr 9	Amplification	p24.2–p22.1	JAK2	known in: BRCA
chr 9	Amplification	p24.2–p22.1	CD274	known in: BCC
chr 8	Amplification	q12.1–q24.3	MYC	known in: BLY; CLL; NB; COREAD; MYMA; PRAD
chr 5	High Amplification	p13.3–q11.2	RICTOR	known in: L
chr 6	Amplification	q16.2–q27	ESR1	known in: UCEC; BRCA; OV
chr 12	Amplification	p13.33–p12.1	CCND2	known in: L
chr 12	Amplification	q14.3–q24.33	FRS2	known in: LIP
chr 14	Amplification	q12–q32.33	FOXA1	Known in: COREAD
chr 14	Amplification	q12–q32.33	PAX9	Known in: NSCLC
chr 14	Amplification	q12–q32.33	NKX2-8	predicted passenger

CGI: Cancer Genome interpreter; PA: Pancreas; BRCA: Breast adenocarcinoma; COREAD: Colorectal adenocarcinoma; BCL: B cell lymphoma; THYM: Thymic; WT: Wilms Tumor; G: Glioma; TH: Thyroid; DSRCT: Desmoplastic small round cell Tumor; PRAD: Prostate Adenocarcinoma; ED: Endometrium; CM: Cutaneous melanoma; L: Lung; OV: Ovary; ALL: Acute Lymphoblastic leukemia; DLBCL: Diffuse Large B cell Lymphoma; MB: Medulloblastoma; AML: Acute Myeloid Leukemia; LK: Leukemia; NF: Neurofibroma; MPN: Malignant Peripheral nerve sheath Tumor; PLEN: Plexiform Neurofibroma; HNC: Head and Neck; SG: Salivary Glands; NSCLC: Non-small lung cancer; BLCA: Bladder; GBM: Glioblastoma multiforme; RB: Retinoblastoma; S: Sarcoma; LIP: Liposarcoma; BCC: Basal cell carcinoma; BLY: Burkitt Lymphoma; CLL: Chronic Lymphocytic Leukemia; NB: Neuroblastoma; MYMA: Myeloma; UCEC: Uterine Corpus Endometroid Carcinoma.

**Table 2:** Somatic mutations present in FaDu Resistant compared with FaDu Parental cell line.

Chromosome	cDNA	Protein	Consequence	Gene	Driver Status
chr 1	c.2492A>T	p.N831I	Missense	EPHA2	Tumor Driver
chr 2	c.2074G>T	p.G692W	Missense	ALK	Tumor Driver
chr 6	c.6341A>G	p.Y2114C	Missense	ROS1	Tumor Driver
chr 6	c.3391A>T	p.K1131*	Nonsense	ZNF292	Tumor Driver
chr 8	c.1648delT	p.S550Qfs*12	Frameshift	UBR5	Tumor Driver
chr 9	c.3127_3129delAGC	p.S1043delS	In Frame Deletion	TSC1	Tumor Driver
chr 9	c.740delC	p.P247Qfs*30	Frameshift	NOTCH1	Tumor Driver
chr 9	c.250_252delGAA	p.E84delE	In Frame Deletion	XPA	Tumor Driver
chr 13	c.3273dupG	p.K1092Efs*233	Frameshift	IRS2	Tumor Driver
chr 14	c.928delG	p.E310Kfs*68	Frameshift	ARID4A	Tumor Driver
chr 15	c.3416delG	p.G1139Efs*25	Frameshift	FANCI	Tumor Driver
chr 16	c.1183delC	p.H395Tfs*78	Frameshift	TRAF7	Tumor Driver
chr 17	c.1420_1422delCAT	p.H474delH	In Frame Deletion	AXIN2	Tumor Driver
chr 19	c.209A>T	p.N70I	Missense	ARHGAP35	Tumor Driver
chr 21	c.146delC	p.P49Qfs*4	Frameshift	RUNX1	Tumor Driver

* Stop codon sequencing.

## Data Availability

The data presented in this study are available upon request from the corresponding author.

## Supplementary Materials

The following supporting information can be downloaded at: https://www.mdpi.com/article/10.3390/cells11010154/s1, Table S1. IC_50_ values for each cetuximab resistant clone established; Table S2. Main changes found in karyotype analysis between Fadu Parental and Fadu Resistant; Table S3. Antibody conditions utilized in Western blot analysis; Supplementary Figure S1. Cetuximab resistant model RTK’S phosphoarray and ROS1 c.6341A>G validation. (A) RTK’s phosphoarray of FaDu parental and FaDu resistant. (B) Integrated Genomics Viewer (IGV) frequency of ROS 1 C.6341A>G mutation in FaDu parental (top) and FaDu resistant (bottom) cells. (C) Sanger sequencing of ROS 1 C.6341A>G mutation in parental and resistant cells. The electropherogram depicts a ROS1 mutation C.6341A>G in the resistant cell line. FaDu Parental; R: FaDu Resistant.
