# Supplementary material for: Comprehensive Molecular Landscape of Cetuximab Resistance in Head and Neck Cancer Cell Lines
Source: Cells. 2022 Jan 4;11(1):154. doi: 10.3390/cells11010154 (PMC8750399; doi:10.3390/cells11010154)
Supplement: Supplementary file 1 [file cells-11-00154-s001.zip › cells-1498716-supplementary.pdf]

Table S1- IC_{50} values for each cetuximab resistant clone established.

Cell line	Fadu Parental	C1	C2	C5	C6	C7	C9	C10
**IC_{50} (µg/mL)**	216,9 ± 0,1	400,4 ± 0,5	1182± 0.4	3722 ± 0,8	572,1± 0,5	3001 ± 1,2	5320± 3,1	ND
**Fold Change**	ND	1,84	5,44	17,1	2,63	13,8	24,5	ND

ND- Not determined

Table S2-Main changes found in karyotype analysis between Fadu Parental and Fadu Resistant.

Cell Line	Karyotype
Parental	51~53,XY,+X,+1,add(1)(q44),+2,add(2)(q37),+3,add(4)(q35),-5,+6,+7,+8,+9,+10,+11,add(11)(p15),+12,-13,add(13)(q34),add(15)(q26),+16,+17,+18,+18,-19,add(19)(q13.4)-20,-21,-22,+3~6mar,1~2min[cp20]
Resistant	52~56,XY,+X,+X,+1,add(1)(p36.3),+2,add(2)(q37)x2,+3,-4,-5,+6,+7,+7,+8,+9,+9,+10,+10,+11,+12,+12,-13,-14,add(14)(q34) +16,+17,+18,-19,-20,-21,-22,+6~9mar,1~2min[cp20]

+ (tadditional chromosome), - (loss of chromosome); p (short arm of chromosome); q (long arm of chromosome); mar (marker chromosome);; add (additional material of unknown origin); cp (composite karyotype)

Table S3- Antibody conditions utilized in western blot analysis.

Antibody	Diluition	Condition	Manufacturer / Code
Anti - β actin	1/1000	4ºC- Overnight	Cell signaling/ #4967
Anti- EGFR	1/1000	4ºC- Overnight	Cell signaling/ #4267
Anti- phospho-EGFR	1/1000	4ºC- Overnight	Cell signaling/ #2234
Anti- phospho- AKT	1/1000	4ºC- Overnight	Cell signaling/ #9271
Anti- AKT (total)	1/1000	4ºC- Overnight	Cell signaling/ #9272
Anti- phospho- p44/42 MAPK	1/1000	4ºC- Overnight	Cell signaling/ #9101
Anti- p44/42 MAPK (total)	1/1000	4ºC- Overnight	Cell signaling/ #9102
Anti-mTOR	1/1000	4ºC- Overnight	Cell signaling/ #2983
Anti-Laminin B1	1/1000	4ºC- Overnight	Cell signaling/ #13435
Anti-FADD	1/1000	4ºC- Overnight	Cell signaling/ #2782
Anti-CD44	1/1000	4ºC- Overnight	Cell signaling/ #37259
Anti-phospho-mTOR	1/1000	4ºC- Overnight	Cell signaling/ # #2974
Anti-E-cadherin	1/1000	4ºC- Overnight	Cell signaling/ #3195
Anti-N-cadherin	1/1000	4ºC- Overnight	Cell signaling/ #13116
Anti- α-Smooth Muscle	1/1000	4ºC- Overnight	Cell signaling/ #19245
Anti-SLUG	1/1000	4ºC- Overnight	Cell signaling/# 9585
Anti-SNAIL	1/1000	4ºC- Overnight	Cell signaling/ #3879
Anti- TGF-β	1/1000	4ºC- Overnight	Cell signaling/#3711
Anti-mouse IgG HPR	1/5000	TA- 1 hour	Cell signaling/ #7076
Anti-rabbit IgG HPR	1/5000	TA- 1 hour	Cell signaling/ #7074

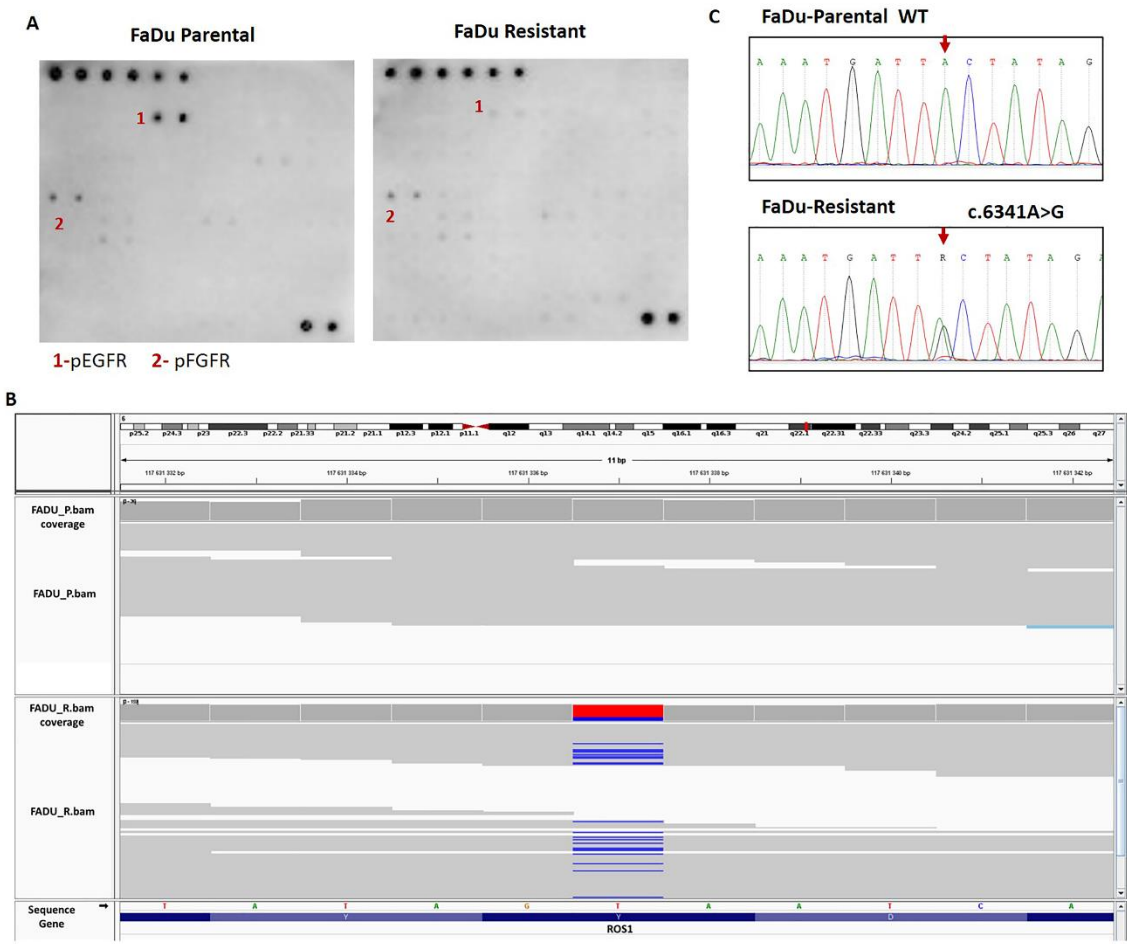

Figure S1- Cetuximab resistant model RTK´S phosphoarray and ROS1 c.6341A>G validation. A) RTK´s phosphoarray of FaDu parental and FaDu resistant. B) Integrated Genomics Viewer (IGV) frequency of ROS 1 C.6341A>G mutation in FaDu parental (top) and FaDu resistant (bottom) cells. C) Sanger sequencing of ROS 1 C.6341A>G mutation in parental and resistant cells. The electropherogram depicts a ROS1 mutation C.6341A>G in the resistant cell line. FaDu Parental; R: FaDu Resistant.
